# Biogeography and taxonomy of extinct and endangered monk seals illuminated by ancient DNA and skull morphology

**DOI:** 10.3897/zookeys.409.6244

**Published:** 2014-05-14

**Authors:** Dirk-Martin Scheel, Graham J. Slater, Sergios-Orestis Kolokotronis, Charles W. Potter, David S. Rotstein, Kyriakos Tsangaras, Alex D. Greenwood, Kristofer M. Helgen

**Affiliations:** 1 Leibniz Institute for Zoo and Wildlife Research, Alfred-Kowalke-Str. 17, 10315 Berlin, Germany; 2 Division of Mammals, Smithsonian Institution, National Museum of Natural History, 10th Street and Constitution Ave, NW, Washington, DC 20560-0108, USA; 3 Department of Paleobiology, Smithsonian Institution, National Museum of Natural History, 10th Street and Constitution Ave, NW, Washington, DC 20560-0108, USA; 4 Department of Biological Sciences, Fordham University, 441 East Fordham Road, Bronx, NY 10458, USA; 5 Marine Mammal Pathology Services, 19117 Bloomfield Road, Olney, MD 20832, USA

**Keywords:** Ancient DNA, extinction, mitochondrial DNA, Panamanian Seaway, Phocidae, systematics

## Abstract

Extinctions and declines of large marine vertebrates have major ecological impacts and are of critical concern in marine environments. The Caribbean monk seal, *Monachus tropicalis*, last definitively reported in 1952, was one of the few marine mammal species to become extinct in historical times. Despite its importance for understanding the evolutionary biogeography of southern phocids, the relationships of *M. tropicalis* to the two living species of critically endangered monk seals have not been resolved. In this study we present the first molecular data for *M. tropicalis*, derived from museum skins. Phylogenetic analysis of *cytochrome b* sequences indicates that *M. tropicalis* was more closely related to the Hawaiian rather than the Mediterranean monk seal. Divergence time estimation implicates the formation of the Panamanian Isthmus in the speciation of Caribbean and Hawaiian monk seals. Molecular, morphological and temporal divergence between the Mediterranean and “New World monk seals” (Hawaiian and Caribbean) is profound, equivalent to or greater than between sister genera of phocids. As a result, we classify the Caribbean and Hawaiian monk seals together in a newly erected genus, *Neomonachus*. The two genera of extant monk seals (*Monachus* and *Neomonachus*) represent old evolutionary lineages each represented by a single critically endangered species, both warranting continuing and concerted conservation attention and investment if they are to avoid the fate of their Caribbean relative.

## Introduction

“… *he discovered a group of islets abounding with sea-fowl and marine animals. On one of them his sailors, in the course of a single night … took fourteen sea-wolves, and killed a vast quantity of pelicans and other birds*.”

Washington Irving (1831)

“*Cruise of Ponce de Leon in search of the Fountain of Youth*”

**Figure 1.**
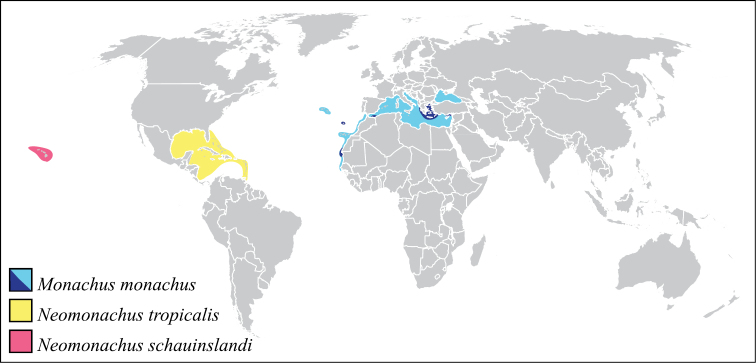
Distributions of the three monk seal species. The range for the Caribbean monk seal is taken from Adam (2004) and is based on documented populations and archeological evidence. The range of the Mediterranean monk seal illustrates both historical (lighter shading) and current (darker shading) distributions.

The Caribbean monk seal, *Monachus tropicalis* (Gray, 1850), first referenced on the New World voyages of Columbus in 1494 and Ponce de Leon in 1513, was one of the few large mammals to become extinct in the twentieth century. Until relatively recently, *Monachus tropicalis* was widely distributed in the Caribbean region (Figure 1), including along the Caribbean coasts of North, Central, and South America, and in the Bahamas and the Greater and Lesser Antilles (Timm et al. 1997, Adam 2004, Adam and Garcia 2003, McClenachan and Cooper 2008). Historical population estimates for the species ranged from 233,000–338,000 prior to the catastrophic decline of the species, caused by unrestricted hunting that increased throughout the nineteenth century (McClenachan and Cooper 2008). No well-documented sightings postdate 1952, and the species is widely regarded as extinct (Mignucci-Giannoni and Odell 2001, Adam and Garcia 2003, McClenachan and Cooper 2008). This is the only historical example of a marine mammal extinction in the tropics, and one of few species-level extinctions of marine mammals in the historical period, along with the Steller’s sea cow (*Hydrodamalis gigas*, North Pacific, last recorded in 1768), the Japanese sea lion (*Zalophus japonicus*, East Asia, last recorded in 1951), and the Yangtze River dolphin or Baiji (*Lipotes vexillifer*, Yangtze River of China, probably extinct within the past decade) (Flannery and Schouten 2001, Wolf et al. 2007, Turvey 2009, Turvey et al. 2007).

There are two species of extant monk seals, both also classified in the genus *Monachus* Fleming, 1822, and both recognized by the IUCN as Critically Endangered species. The Mediterranean monk seal, *Monachus monachus* (Hermann, 1779), occurred historically throughout the Mediterranean and Black Seas and along the Atlantic Coast of northern Africa (Figure 1). Today, a heavily fragmented global population of 350–450 individuals remains, dispersed throughout the Mediterranean and south-eastern North Atlantic (Sergeant et al. 1978, Pastor et al. 2007, Aguilar and Lowry 2013). The Hawaiian monk seal, *Monachus schauinslandi* Matschie, 1905, occurs throughout the Hawaiian island chain (Figure 1), with a current population of about 1000 animals (Lowry and Aguilar 2008, Schultz et al. 2009). Both of these extremely endangered species are threatened by many anthropogenic stressors, including mortality from hunting, entanglement in fishing gear, competition with fisheries for food, loss and disturbance of coastal habitats and breeding sites, oceanic pollution, and emerging diseases (Lowry and Aguilar 2008, Aguilar and Lowry 2013, Kovacs et al. 2012).

The relationship of the Caribbean monk seal to the two living species of *Monachus* has long been of interest to paleontologists, evolutionary biologists, and biogeographers (Repenning and Ray 1977, Wyss 1988, Bininda-Emonds and Russell 1996, Bininda-Emonds et al. 1999, Higdon et al. 2007). Morphological assessments have, to date, remained equivocal with regard to the relationships of *Monachus tropicalis* to the other monk seals. King (1956) was the first to compare the morphology of all three monk seal species in detail (though Matschie [1905] reported important earlier observations). Although King did not explicitly state a hypothesis regarding phylogenetic relationships within the genus, she noted a greater similarity between the skulls and dentitions of the Caribbean and Hawaiian monk seals than between either of these species and the Mediterranean monk seal (King 1956, King and Harrison 1961; also Kenyon and Rice 1959). Rice (1998) argued the opposite, suggesting greater similarity between the Mediterranean and Caribbean species. Quantitative cladistic analyses have suggested sister-group relationships for the Caribbean monk seal to both the Mediterranean monk seal (Koretsky and Grigorescu 2002) and the Hawaiian monk seal (Bininda-Emonds and Russell 1996) on the basis of cranial and postcranial anatomy. Wyss’s (1988) analysis of phocid morphology even suggested paraphyly of the genus, recovering ‘*Monachus*’ as a basal grade of phocids with ‘*Monachus*’ *schauinslandi* as the sister taxon to all other extant phocids. More recently, molecular phylogenetic analyses have confirmed monk seal monophyly, at least for the extant species, and their position in Monachinae as the likely sister group to *Mirounga* and the lobodontine seals of the southern oceans (Arnason et al. 1995, Ledje and Arnason 1996, Davis et al. 2004, Fyler et al. 2005, Fulton and Strobeck 2010a, 2010b). However, because the Caribbean monk seal is extinct and represented only by historical museum specimens (skins and skeletal material), rather than fresh frozen tissues, previous molecular phylogenies of phocids have not included the species. This has resulted in ambiguity regarding broader questions in pinniped evolution, such as the place of origin of the genus *Monachus* and the subfamily Monachinae (Ray 1976a, Deméré et al. 2003, Fyler et al. 2005, Fulton and Strobeck 2010a).

The initial objective of this study was to determine the phylogenetic placement of *Monachus tropicalis* and to estimate the timing of its divergence from the Hawaiian and Mediterranean monk seals, as well as from other phocids. Using ancient DNA methods (Willerslev and Cooper 2005), we successfully extracted and amplified a complete *cytochrome b* (hereafter *cyt*b) sequence from three *Monachus tropicalis* museum skins housed in the National Museum of Natural History, Smithsonian Institution (formerly United States National Museum, abbreviated USNM) and subjected these to phylogenetic analyses. Based on these results, as well as detailed examination of monk seal cranial morphology and considerations of divergence and biogeography, we recognize and name a new genus, *Neomonachus*, for the Caribbean and Hawaiian monk seals.

## Materials and methods

### Specimens examined

We examined all specimens of the three *Monachus* species in the collections of the USNM, which houses the only large collection of *Monachus tropicalis* (44 specimens, mostly represented by skins and/or skulls, most of which were collected by E.W. Nelson and E.A. Goldman at the turn of the twentieth century; Goldman 1951). The USNM also holds substantial material of *Monachus schauinslandi* for comparative study (54 specimens), but more limited material of *Monachus monachus* (one adult skull, likely a male, of unknown provenance, and one juvenile skull). We supplemented our observations of *Monachus* by studying material housed in the mammalogy collections of the Field Museum of Natural History, Chicago (FMNH), and the American Museum of Natural History, New York (AMNH) (including three skulls of *Monachus monachus*), and drawing from observations on specimens in the Natural History Museum (London) (mainly *Monachus monachus*) as reported by King (1956). Dental measurements were collected by GJS using a Mitutuyo^©^ digital caliper with 0.01 mm precision. Specimens shown in the photographic figures are USNM 219059 (*Monachus monachus*), USNM 181250 and 395761 (*Monachus schauinslandi*), and USNM 102534 and 102536 (*Monachus tropicalis*).

### Samples, ancient DNA extraction, PCR and DNA sequencing

Several grams of dry skin were collected from six Caribbean monk seal museum specimens. All were originally collected in the Triangle Keys, a remote and tiny group of islands in Campeche, off the Yucatan Coast of Mexico. Two specimens (USNM 83711 and 83712) from the Triangle Keys were originally kept as captive animals on display at the National Zoological Park (Washington, D.C.), and transferred to the museum upon their death in 1897. The remaining specimens were collected by E.W. Nelson and E.A. Goldman, in June 1900 (Goldman 1951). Specimen catalog numbers and other information are provided in Table 1.

**Table 1.** Specimen information for Caribbean monk seals sampled in this study.

USNM Number	Sex	Locality	Year	Collector
83711	Female	Bay of Campeche, Mexico	1897	National Zoological Park
83712	Male	Bay of Campeche, Mexico	1897	National Zoological Park
100358	Male	Triangle Keys, Bay of Campeche, Mexico	1900	EW Nelson and EA Goldman
100359	Female	Triangle Keys, Bay of Campeche, Mexico	1900	EW Nelson and EA Goldman
102527	Female	Triangle Keys, Bay of Campeche, Mexico	1900	EW Nelson and EA Goldman
102535	Female	Triangle Keys, Bay of Campeche, Mexico	1900	EW Nelson and EA Goldman

Historical DNA experiments were conducted in an ancient DNA laboratory dedicated to extractions and other pre-PCR procedures involving museum samples. The laboratory was equipped with plexiglass UV PCR hoods for extraction and PCR setup. All reagents and equipment in the lab were exclusively dedicated to ancient DNA experimental use. Modern DNA and PCR products never entered the room. All workstations were regularly UV-irradiated to avoid contamination. Extractions were performed with the Geneclean Kit for Ancient DNA (MP Biomedicals) as described by Roca et al. (2009). Primers for eleven PCR products ranging in size from 48–549 basepairs (bp) were designed to cover the 1140 bp of the monk seal *cyt*b gene (primers are shown in Table 2, amplicon positions are shown in Suppl. material 1). PCR amplicons were produced by conventional and multiplex PCR. One extraction blank and one PCR water negative control were carried out for each PCR experiment. Conventional PCR amplifications were performed in 30.5 μl volumes containing 1 μl of 25 mg/ml BSA (Fermentas), 1 μl of 10 pmol forward primer, 1 μl of 10 pmol reverse primer, 22.5 μl of Platinum PCR SuperMix High Fidelity Taq polymerase (Invitrogen), and 5 μl of purified sample DNA. The thermal cycling conditions were 94 °C for 4 min, followed by 60 cycles at 94 °C for 30 s, at 55 °C for 30 s, and at 72 °C for 30 s, then finally 72 °C for 5 min. Multiplex PCR amplifications were carried out in 59.5 μl volumes that included 2 μl of 25 mg/ml BSA, 7.5 μl of primer mix with primers of 1 pmol/μl concentration each, 45 μl of Platinum PCR SuperMix High Fidelity Taq polymerase, and 5 μl of purified sample. The PCR temperature profile was the same as in conventional PCR amplifications except that only 25 cycles were performed. Products of multiplex PCR were purified using QIAquick PCR purification kits (Qiagen) according to manufacturer instructions. Amplification of multiplex PCR products used the same cycling conditions as conventional PCR amplification except that 1 μl of purified multiplex product was used as template. Amplification products were separated by electrophoresis on 3% agarose gels stained with GelRed (Biotium). Correct size products were cloned using the pGEM®-T Vector Systems Kit (Promega) and JM109 High Efficiency Competent Cells (Promega) following manufacturer instructions. Ten clones of each PCR product were chosen and used in a colony PCR performed as described by Roca et al. (2009). PCR products from insert positive colonies were purified using QIAquick PCR purification kits and Sanger sequenced using standard M13 forward and reverse primers (StarSeq GmbH, Berlin). Aligned consensus sequences are shown for *cyt*b in Suppl. material 2. Consensus sequences, generated from 3-5 clones per amplicon from 2-3 replicate amplicons covering each base, were deposited in GenBank (accession JX853967). To enable a limited assessment of genetic variability within the Triangle Keys population prior to extinction, D-loop hypervariable region sequences were determined. Two fragments of the hypervariable region were successfully amplified and sequenced from 3 of the 6 extracted seals (Suppl. material 3).

**Table 2.** List of primers used in this study. Asterisks indicate that the sequence extends over the 3’ or 5’ ends of the *cytb* gene. Amplicon sizes are shown in base pairs. The primer combinations are designated by numbers, e.g. P7.

Primer	Name	Sequence (5’–3’)	Length (bp)
P7	CYTB-MT-F1 CYTB-MT-R1	ATG ACC AAC AT(C/T) CGA AAA AC AAA GGC TGT (A/G)GT TGT GTC TG	149
P10	CYTB-MT-F4 CYTB-MT-R4	(T/C)TA CCA TGA GGA CAA AT(G/A) TC TG(T/G) ACT GCT (A/G)CT AGT GCT	153
P16	CYTB-MT-F1 CYTB-MT-R4	ATG ACC AAC AT(C/T) CGA AAA AC TG(T/G) ACT GCT (A/G)CT AGT GCT	549
P20	Flank-MT-F CYTB-FFla-R	CCA CCG TTG TAA TTC AAC TA GAT GAG TGA GTT ATT GAT AA	48*
P21	CYTB-MT-F9 CYTB-MT-R9	TAT TCC TAG CTA TAC ACT AC GTG AAT GTG TAG GAG CCG TA	144
P22	CYTB-MT-F10 CYTB-MT-R10	TAT CTG CTT ATA TAT ACA CGT A AGT AGA TTG GTG ATG ACG GT	135
P23	CYTB-MT-F11 CYTB-MT-R11	CAC TTC ATT ATA CCC TTC AT AAT GGG ATT TTG TCT GAG T	77
P24	CYTB-MT-F11 CYTB-MT-R5	CAC TTC ATT ATA CCC TTC AT CC(C/T) AGA ATG TCT TTA ATT GT	109
P32	CYTB-MT-F13 CYTB-MT-R13	CTC AGA CAA AAT CCC ATT TC GGG TTT GAT ATG TGG TGG	131
P40	CYTB-MT-F11 CYTB-MT-R13	CAC TTC ATT ATA CCC TTC AT GGG TTT GAT ATG TGG TGG	229
P42	CYTB-MT-F16 Flank-MT-R3	AGA CCC TGA CAA CTA TAC C GGT CTT GTA AAC CAA AAA CG	413*

### Phylogenetic analysis

We integrated the Caribbean monk seal *cyt*b sequence into an alignment of 35 previously sequenced caniform carnivore *cyt*b sequences containing all extant phocid species (*n* = 18) and 17 outgroup taxa spanning 5 families (Table 3). We performed thorough phylogenetic analyses under the Maximum Likelihood (ML) optimality criterion using the POSIX-threads build of RAxML v7.5.3 (Stamatakis 2006) by running 100 searches starting from independent stepwise-addition maximum parsimony starting trees and the general time-reversible (GTR) substitution model with among-site rate heterogeneity modeled through the Γ distribution and four discrete rate categories (Lanave et al. 1984, Yang 1994). Node support was estimated with 1000 bootstrap pseudoreplicates (Felsenstein 1985) and the Shimodaira-Hasegawa-like nonparametric approximate likelihood-ratio test (a.k.a. SH-aLRT) (Shimodaira and Hasegawa 1999, Guindon et al. 2010). Bayesian inference of phylogeny was performed using MrBayes v3.2 (Ronquist et al. 2012). We performed two simultaneous runs of Metropolis-coupled Markov chain Monte Carlo each with four chains (one cold, three heated) of 10 million generations, sampling from the posterior distribution every 1000 steps. We ensured that both runs had converged on the target distribution and that adequate effective sample sizes had been obtained using Tracer v1.5. To determine the most appropriate partitioning scheme, we performed three separate analyses: 1) an unpartitioned analysis; 2) a two-partition analysis, with codon positions 1 and 2 treated as the first partition and the third codon position as the second partition; and 3) a three-partition analysis where each codon position was assigned to a distinct partition. Each partition was assigned a GTR + I + Γ model. Based on Bayes Factor comparison using the method of Suchard et al. (2001) implemented in Tracer (http://tree.bio.ed.ac.uk/software/tracer), the three-partition scheme received decisive support, *sensu* Kass and Raftery (1995), over the two-partition (log_10_BF = 23.24) and unpartitioned (log_10_BF = 277.6) schemes. Finally, we performed a maximum parsimony analysis (MP) using the DNAPARS program of the PHYLIP v3.6 package (Felsenstein 2005). All sites were treated as unweighted. Node support was evaluated through 1000 bootstrap pseudo-replicates mapped on a majority rule consensus tree.

**Table 3.** List of species and associated GenBank accession information used in phylogenetic analysis of *cytb* sequence data.

Family	Binomial	Common name	GenBank accession no.	Source
Phocidae	*Phoca largha*	Spotted seal	X82305	Arnason et al. (1995)
	*Phoca vitulina*	Harbor seal	X82306	Arnason et al. (1995)
	*Pusa sibirica*	Baikal seal	AY140977	Palo and Vainola (2006)
	*Pusa hispida*	Ringed seal	X82304	Arnason et al. (1995)
	*Pusa caspica*	Caspian seal	AY140978	Palo and Vainola (2006)
	*Halichoerus grypus*	Gray seal	NC001602	Arnason and Gullberg (1993)
	*Cystophora cristata*	Hooded seal	X82294	Arnason et al. (1995)
	*Histriophoca fasciata*	Ribbon seal	X82302	Arnason et al. (1995)
	*Pagophilus groenlandicus*	Harp seal	X82303	Arnason et al. (1995)
	*Erignathus barbatus*	Bearded seal	AY170104	Yoder et al. (2003)
	*Hydrurga leptonyx*	Leopard seal	AY377323	Davis et al. (2004)
	*Leptonychotes weddellii*	Weddell seal	AY377324	Davis et al. (2004)
	*Ommatophoca rossii*	Ross seal	AY377322	Davis et al. (2004)
	*Lobodon carcinophagus*	Crabeater seal	AY377321	Davis et al. (2004)
	*Neomonachus schauinslandi*	Hawaiian monk seal	X72209	Arnason and Gullberg (1993)
	*Neomonachus tropicalis*	Caribbean monk seal	JX853967	this study
	*Monachus monachus*	Mediterranean monk seal	AY377327	Davis et al. (2004)
	*Mirounga leonina*	Southern elephant seal	AY377326	Davis et al. (2004)
	*Mirounga angustirostris*	Northern elephant seal	AY377325	Davis et al. (2004)
Otariidae	*Arctocephalus australis*	South American fur seal	AY377329	Davis et al. (2004)
	*Arctocephalus forsteri*	New Zealand fur seal	X82293	Arnason et al. (1995)
	*Arctocephalus gazella*	Antarctic fur seal	X82292	Arnason et al. (1995)
	*Otaria flavescens*	South American sea lion	AY377328	Davis et al. (2004)
	*Zalophus californianus*	California sea lion	X82310	Arnason et al. (1995)
	*Eumetopias jubatus*	Steller sea lion	DQ145021	Harlin-Cognato et al. (2006)
	*Callorhinus ursinus*	Northern fur seal	NC008415	Arnason et al. (2006)
Odobenidae	*Odobenus rosmarus*	Walrus	X82299	Arnason et al. (1995)
Mustelidae	*Meles meles*	Eurasian badger	NC011125	Arnason et al. (2007)
	*Gulo gulo*	Wolverine	NC009685	Arnason et al. (2007)
	*Mustela nivalis*	Least weasel	HM106319	Yu et al. (2011)
Ursidae	*Ursus americanus*	American black bear	NC003426	Delisle and Strobeck (2002)
	*Ursus arctos*	Brown bear	HQ685963	Keis et al. (2013)
	*Ailuropoda melanoleuca*	Giant panda	NC009492	Peng et al. (2007)
Canidae	*Vulpes vulpes*	Red fox	NC008434	Arnason et al. (2006)
	*Nyctereutes procyonoides*	Raccoon dog	GU256221	Chen and Zhang (2012)
	*Canis lupus*	Gray wolf	AY170103	Yoder et al. (2003)

### Divergence time estimation

We estimated divergence times using BEAST v1.7.4 (Drummond et al. 2012). Fulton and Strobeck (2010a) noted that phocid divergence time estimates derived from mitochondrial sequences alone resulted in older ages, in some cases as much as 5 million years older, than were estimated from nuclear only or nuclear plus mitochondrial data. Such an artifact is problematic for our purposes because such dramatic differences in divergence time estimates will result in substantially different interpretations of the biogeographic context for Caribbean monk seal evolution. For the purposes of divergence dating in this study, we therefore integrated our Caribbean monk seal *cyt*b sequence into the alignment of 15 nuclear genes plus complete mitochondrial genome sequence data from Fulton and Strobeck (2010a), kindly provided to us by Tara Fulton. GenBank accession numbers for sequences used are listed in Appendix S1 of Fulton and Strobeck (2010a). Prior to adding the Caribbean monk seal sequence, we removed the third codon position, which Fulton and Strobeck (2010a) found to show a saturated substitution rate in their analyses, thus having the potential to overestimate branch lengths. The alignment was partitioned by nuclear gene, and all mitochondrial sequences (codon positions 1 and 2) treated as a single, additional partition. Substitution models followed those determined through Akaike Information Criterion (Akaike 1974) comparison in MrModelTest (https://github.com/nylander/MrModeltest2) by Fulton and Strobeck (2010a), and specified in Appendix S2 of Fulton and Strobeck (2010a).

Some authors have questioned the appropriateness of using taxa with incomplete sequences in phylogenetic and divergence time inferences (Lemmon et al. 2009), noting that doing so can result in misleading topological and branch length estimates (but see Roure et al. 2013 for a comment on this simulation study). However, a number of other studies have suggested that even highly incomplete taxa can have a positive influence in phylogenetic analysis (e.g., Huelsenbeck 1991, Wiens and Moen 2008, Wiens and Morrill 2011, Wiens and Tiu 2012). Ideally, we would have sequenced at least one nuclear locus for our Caribbean monk seal sample for inclusion in phylogenetic analysis. However, due to the sequence divergence from other seals and relatively poor quality of the DNA extracted, this would represent a major undertaking that would have a high chance of failure by PCR-based approaches from 100 year-old museum samples and would be unlikely to alter the conclusions of our study. Ultimately, we opted to include the Caribbean monk seal *cyt*b sequence in a larger pinniped alignment of nuclear and mitochondrial sequence data for two reasons. First, accurate divergence time inference is key to understanding the biogeographic context for the evolution of New World monk seals. Given the results presented by Fulton and Strobeck (2010a), we simply cannot rely on divergence time estimates from one mitochondrial locus alone, across all pinnipeds, for a sound evolutionary interpretation. Second, previous studies suggest that incomplete sequences do not have a substantial impact on branch length estimation when the model of sequence evolution is correctly specified (Wiens and Moen 2008), and that they are less problematic when the branch leading to the sparsely sampled taxon is relatively short (Roure et al. 2013). Because our divergence time analysis, like that of Fulton and Strobeck (2010a), uses a relaxed, uncorrelated molecular clock, with substitution models unlinked across loci, the impacts of the large amount of missing data for the Caribbean monk seal should be limited. In contrast, the benefit of our approach is that it effectively uses divergence times for extant phocids inferred from the more complete nuclear + mitochondrial alignment as a framework for constraining the range of possible divergence time estimates for the Caribbean monk seal from its closest living relatives.

We performed joint estimation of topology and divergence times in BEAST v1.7.4 (Drummond et al. 2012) by using the same set of node priors as in Fulton and Strobeck (2010a; see their Table 2, with reasoning in the supplementary information) but with two additions: a prior for crown Canidae was applied to the node uniting the most recent common ancestor of *Canis lupus* and *Vulpes lagopus*, and a prior for the first appearance of *Eumetopias* and *Zalophus* was applied to the node uniting *Eumetopias jubatus* and *Arctocephalus* spp., following ages and justifications provided in Slater et al. (2012). We ran two independent Markov chains for 50 million generations, sampling from the posterior distribution every 10,000 generations. After visually checking for convergence of the chains in Tracer, we conservatively removed the first 25% of samples as burn-in and produced a maximum clade credibility tree from the retained sample.

### Sequence divergence among phocids

Fulton and Strobeck (2010b) noted that pairwise genetic distances between the two extant monk seal species were greater than the distances between any pair of species within extant genera. They further observed that the magnitude of genetic divergence between the two monk seals was more comparable to tribal-level differences among other phocids. To further examine variation in sequence divergence within *Monachus* and among phocid species in general, we replicated their analyses incorporating our Caribbean monk seal sequence. Following the same approach taken by Fulton and Strobeck (2010b), we computed logdet pairwise genetic distances from the aligned phocid *cyt*b sequences and summarized values at interspecific, generic, and tribal levels within Phocinae and Monachinae. Distances were computed using the dist.dna() function in the APE (Paradis et al. 2004) package for R (R Development Core Team 2012).

## Results

### Sequence retrieval

The Caribbean monk seal *cyt*b gene was generated from a 112-year-old specimen (USNM 100358) from multiple extractions and overlapping PCR amplicons (Suppl. material 1). The consensus sequence of the 1140 bp of *cyt*b represents at least duplicate coverage of every base by an independent amplicon. The results were consistent across all experiments, except for two nucleotide positions that differed in more than one PCR. Those were G-A changes and are most likely due to template deamination on the complementary strand (Hofreiter et al. 2001). The correct base at the two positions was resolved with further independent PCRs that indicated that at both positions, G was the correct nucleotide, as A only appeared among clones in one amplicon. The consensus sequence displayed 90% identity to that of *Monachus schauinslandi* and high similarity to other phocid sequences. The fact that the overlapping sequences used to create the consensus sequence matched in the overlaps and no premature stop codons were identified in any amplicon supports the conclusion that the *cyt*b sequence represents organellar mtDNA and not a nuclear-derived mtDNA sequence (Bensasson et al. 2001). The hypervariable region sequences determined from three Caribbean monk seal individuals were identical for both non-overlapping fragments (Suppl. material 3), possibly indicating limited genetic diversity in one of the last surviving populations of the species at the turn of the twentieth century.

### Phylogenetic analysis

**Figure 2.**
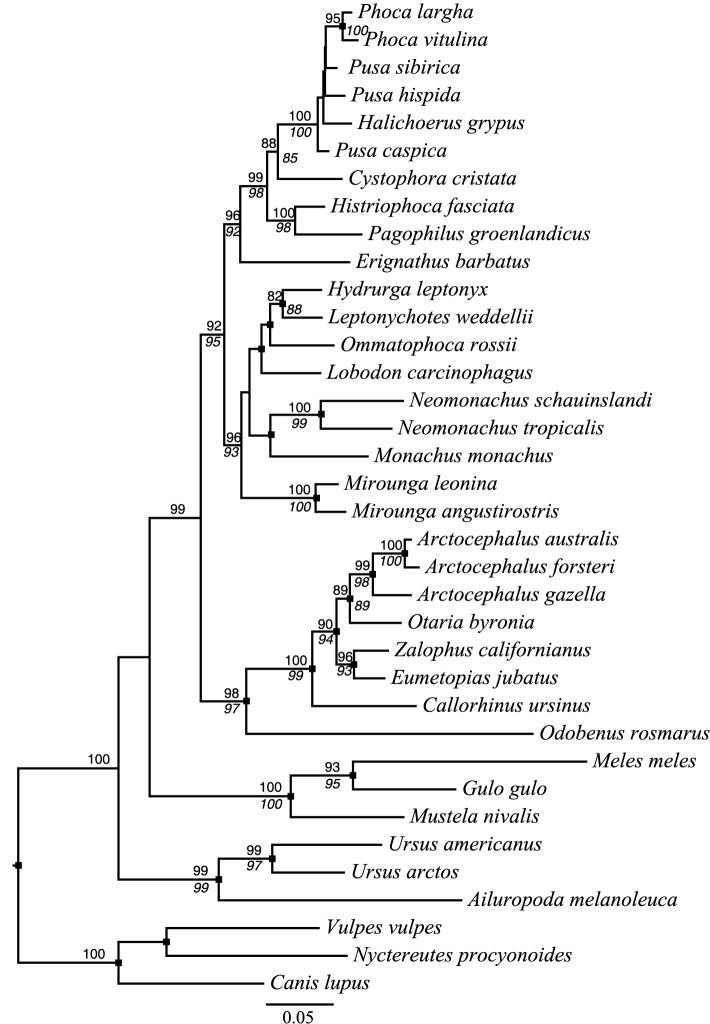
Maximum likelihood phylogram inferred from *cytb* sequence data using the GTR + Γ_4_ substitution model. Node support is expressed as the percent proportion of 1000 bootstrap pseudoreplicates that agree with the bipartitions on the best ML tree (above internode branches) as well as the aLRT SH-like score (below internode branches). Support values above 80% for both measures are shown. Black boxes indicate nodes recovered with >0.88 posterior probability in Bayesian analyses. The scale bar indicates the number of substitutions per site.

Phylogenetic analyses of the complete *cyt*b sequences under MP, ML, and BI (Figure 2, Suppl. material 4) recovered a monophyletic monk seal clade (BS-MP = 45%, BS-ML = 70%, SH-aLRT = 0.78, PP = 0.93), with a well-supported subclade of New World species (BS-MP = 96%, BS-ML = 100%, SH-aLRT = 0.99, PP = 1.00). Our analyses therefore indicate that the Caribbean and Hawaiian monk seals are more closely related to each other than either is to the Mediterranean monk seal, supported by 9 synapomorphic, non-synonymous changes. The rest of our *cyt*b tree topology is generally consistent with earlier studies of phocid relationships. One notable exception concerns relationships at the base of Monachinae, where we recovered *Mirounga* (the elephant seals) as the sister lineage to other monachines (Figure 2). Recent studies utilizing both nuclear and mitochondrial loci have revealed that *Monachus* is sister to *Mirounga* + Lobodontini (Fyler et al. 2005, Fulton and Strobeck 2010a, 2010b). The (*Mirounga*, (Monachini, Lobontini)) relationship is weakly supported in our analyses, however (BS-ML = 55%, SH-aLRT = 0.73, PP = 0.8), and likely reflects inadequacy of *cyt*b data alone to resolve deeper, rapid divergence events (Fyler et al. 2005). The more common branching order of (Monachini, (*Mirounga*, Lobontini)) was recovered in our parsimony analyses (Suppl. material 4), but with even weaker support (BS-MP = 29%).

### Divergence time estimation

**Figure 3.**
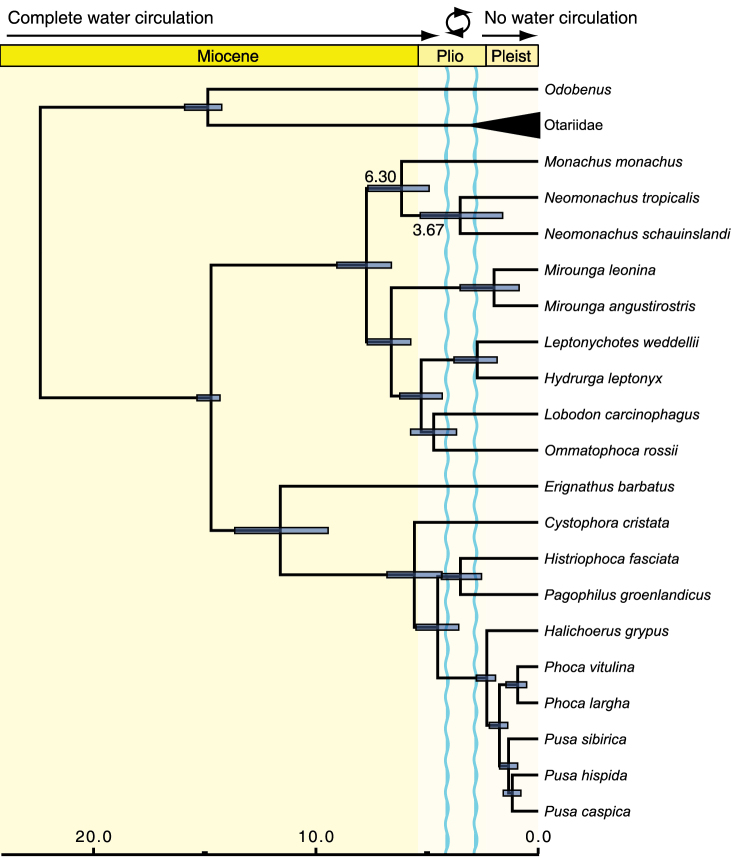
Time-calibrated phylogeny of the seals estimated from combined nuclear and mitochondrial data. Time scale is in millions of years before present. Note that the chronogram has been pruned to show only true seals and immediate pinniped outgroups. Node bars show the 95% HPD intervals for divergence time estimates and mean ages are labeled for the two divergence times within the monk seals. Labeling at the top indicates water circulation through the Central American Seaway, the circle and associated wavy blue lines indicate a period during which water circulation periodically ceased and resumed but a shallow seaway remained open.

We estimated the divergence of the Caribbean and Hawaiian monk seals at 3.67 Mya (95% HPD = 1.90–5.45 Mya). Node age estimates throughout the rest of the chronogram derived from analysis of the nuclear + mitochondrial genome data are consistent with those recovered by the original analysis of Fulton and Strobeck (2010a) (Figure 3). The Mediterranean-New World monk seal divergence is dated at 6.30 Mya (95% HPD = 4.98–7.64 Mya) in our analyses, slightly older than, but broadly overlapping with the age of 5.48 Mya (95% HPD = 3.93–7.13 Mya) reported by Fulton and Strobeck (2010a).

### Sequence divergence

**Figure 4.**
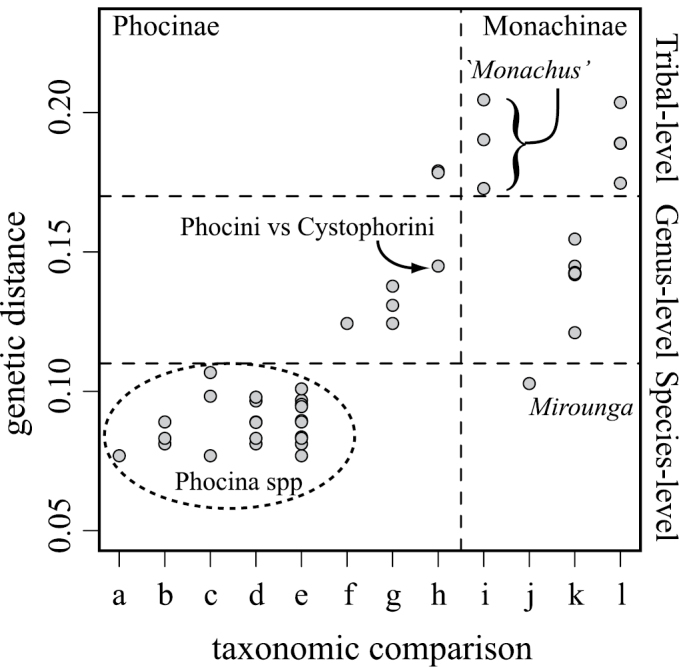
Genetic distances between currently recognized taxonomic units within Phocidae derived from logdet distances for *cytb*. Distances within: **a** *Phoca* **b** *Pusa* **c** *Phoca* versus *Halichoerus* **d** *Pusa* versus *Halichoerus* **e** *Phoca* versus *Pusa* **f** *Histriophoca* versus *Pagophilus* **g** Phocini **h** Phocinae **i** *Monachus* **j** *Mirounga* **k** Lobodontini, and **l** Monachini.

Pairwise genetic distances incorporating the Caribbean monk seal (Figure 4) confirm the findings of Fulton and Strobeck (2010b). Genetic distances between currently recognized generic lineages within the subtribe Phocina (*Phoca*, *Pusa*, and *Halichoerus*) are more similar to species-level, rather than generic-level, distinctions in other phocid lineages (perhaps an indication that this lineage is generically ‘oversplit’). Within *Monachus*, our analyses reveal that sequence divergence between all three species is of a similar magnitude and equivalent to tribal-level divergence in other phocine and monachine taxa.

## Discussion

### Systematics and evolution of the Caribbean monk seal

Our results provide the first molecular evidence for the phylogenetic placement of the Caribbean monk seal. The monophyly of *Monachus* has been questioned on the basis of morphology (Wyss 1988), but molecular studies, which have not included the Caribbean monk seal, have confirmed the sister relationship of the two extant monk seal species (Arnason et al. 2006, Ledje and Arnason 1996, Davis et al. 2004, Fyler et al. 2005, Fulton and Strobeck 2010a, 2010b). Our analyses confirm the monophyly of *Monachus*, albeit with relatively deep divergences among all three Recent species (see below).

The exact relationships of the extinct Caribbean monk seal have previously been unclear, with sister relationships to both extant monk seal species suggested on the basis of morphology (Bininda-Emonds et al. 1999, Koretsky and Grigorescu 2002). Our finding of a sister relationship between the Caribbean and Hawaiian monk seals, and our estimate for their associated divergence time, have important implications for understanding the biogeographic context of monachine evolution. Extant monachine seals are primarily Southern Ocean specialists, with some species, including members of *Monachus*, extending to tropical and mid-latitude portions of the Atlantic and Pacific. This has led to some confusion regarding the origin of the subfamily as a whole, as well as the processes giving rise to the disjunct distribution of the recent species of *Monachus* (e.g., Fyler et al. 2005, Fulton and Strobeck 2010a). Our results suggest that an eastern mid-Atlantic origin for *Monachus* with subsequent dispersal to the western Atlantic in the common ancestor of the two New World species is plausible (e.g., Deméré et al. 2003). However, a western Atlantic origin with dispersal to the eastern Atlantic in the lineage leading to *Monachus monachus* would be equally parsimonious based on our results, and attempts to quantitatively assess these hypotheses using Dispersal-Extinction-Cladogenesis models (Ree and Smith 2008, data not shown) were equivocal regarding the center of origin of *Monachus*, Monachinae, and Phocidae. Fortunately, monachines possess a rich fossil record from Middle Miocene and Pliocene deposits of Europe (Koretsky and Grigorescu 2002), the northwestern Atlantic (Ray 1976a, Deméré et al. 2003), and the Pacific coast of South America (Walsh and Naish 2002, Valenzuela-Toro et al. 2013, Amson and Muizon 2013). Ray (1976a, 1976b) noted that monachines were the dominant pinnipeds of the North Atlantic until the Pliocene, and suggested that the distribution of extant monk seals could be explained by extinction of high latitude species and their replacement by phocine seals. Integration of fossil monachines into the complete molecular phylogeny of Recent phocids that we have generated here will undoubtedly play an important role in robustly resolving these biogeographic questions (e.g., Wood et al. 2013).

Our analyses confirm that the closure of the Central American seaway after the completion of the Panamanian Isthmus could have played a prominent role in explaining the evolution and distributions of the two New World monk seals. Although the phylogenetic placement of the Caribbean monk seal has been uncertain until now, this significant geological and biotic event has traditionally been invoked to explain the divergence of New World *Monachus* species through vicariance (e.g., Ray 1976a, Repenning and Ray 1977, Repenning et al. 1979, Deméré et al. 2003, Fyler et al. 2005, Fulton and Strobeck 2010a). Exact dates for the final formation of the Panamanian land bridge, which led to the Great American Biotic Interchange in terrestrial ecosystems, are uncertain but estimates usually range from about 4–2 Mya (e.g., Coates et al. 1992, Coates and Obando 1996, Bartoli et al. 2005, Jackson and O’Dea 2013; though see also Montes et al. 2012). Oxygen isotope data from the foraminiferal record provide valuable information about oceanic salinity levels on either side of the forming land bridge prior to this time and, by extension, indicate the degree of water flow and connectivity between the Pacific and Atlantic oceans (Haug et al. 2001) that would be essential for dispersal of marine mammal populations. Divergence in estimated salinity levels from Pacific and Caribbean sites indicates that intermediate water transfer from the Pacific to the Atlantic began to reduce approximately 4.5–4.0 Mya, but that a shallow surface water connection remained, causing low-salinity Pacific surface waters to flow into the Caribbean sea, until at least 3.0 Mya (Haug et al. 2001, Bartoli et al. 2005). Convergence in foraminiferal δ^18^O values at 3.3 and 3.8 Mya further indicate that intermediate water transfer resumed, albeit temporarily, during this window (Bartoli et al. 2005), leading to full potential for dispersal between Atlantic and Pacific Oceans by marine mammals. We estimated a mean divergence time for the two New World monk seals of 3.67 Mya, well within this period of reduced connectivity between the Pacific and Atlantic (Figure 3), suggesting that vicariance is a viable explanation for the divergence of the two species. The common ancestor of New World monk seals may have been more broadly distributed throughout the shallow Central American Seaway during the Late Pliocene, although to the best of our knowledge there is no fossil evidence for the presence of *Monachus* along the Pacific shoreline. Populations of this ancestral form would likely have also used small islands that ultimately became part of Panama as haul-out sites. The final closure of the Seaway at 2.5–2.0 Mya created allopatric populations, split between the two oceans, that would give rise to the modern Caribbean and Hawaiian species. The upper 95% HPD for the age of the most recent common ancestor of *Monachus tropicalis* and *Monachus schauinslandi* in our analyses was 1.90 Mya, suggesting that divergence postdating the closure of the seaway (e.g., via dispersal around the southern tip of South America) is unlikely.

### Generic taxonomy: definition of a second monk seal genus

Though all three monk seals are currently classified in a single genus, *Monachus*, the split between New World monk seals and the Mediterranean monk seal is far older than the basal divergence within any other currently recognized modern seal genus (Figure 3) and genetic distances between *Monachus* species exceed those among other phocid tribes (Figure 4). Wyss (1988) advocated for the splitting of *Monachus* into multiple genera once the relationships among monk seals were better resolved. Although our examination of specimens did not reveal sufficient characters to warrant supra-specific distinction between the Caribbean and Hawaiian monk seals, we did find a large number of characters differentiating the two New World monk seals from the Mediterranean monk seal. Tying together the genetic, temporal, and morphological evidence, we here propose a new genus for the New World species.

All previous generic-level names applied to monk seals have as their type species the Mediterranean monk seal, *Monachus monachus* (Hermann, 1779), and are thus synonyms of *Monachus* Fleming, 1822, the earliest generic name erected with that species as its type. These synonyms include *Pelagios* F. Cuvier, 1824 (including its various subsequent spellings and the replacement name *Rigoon* Gistel, 1848), *Pelagocyon* Gloger, 1841, and *Heliophoca* Gray, 1854, as well as Herrera’s (1899) eccentric (and invalid) usage of *Mammonachus* (Gray 1866, Thomas 1895, Palmer 1904, King 1956, Wozencraft 2005). Neither the Caribbean nor the Hawaiian monk seal has been designated as the type species of any previously erected genus-level name, so a new generic name is required.

#### Neomonachus

Slater & Helgen gen. n.

http://zoobank.org/1F643A9A-4D26-44DD-B7D7-C3EB9BE3804B

http://species-id.net/wiki/Neomonachus

##### Type species.

*Monachus schauinslandi* Matschie, 1905 (endemic to the Hawaiian Islands).

##### Other included species.

A second species, *Monachus tropicalis* (Gray, 1850) (endemic to the Caribbean region, recently extinct). We note here, as an aside, that an earlier specific epithet, *antillarum* Gray, 1849, has often been included in the synonymy of *tropicalis*, where it is identified either as a partial synonym (e.g., Allen 1880: 708, 1887: 3, Adam 2004: 1) or a *nomen nudum* (Wozencraft 2005: 598), or simply listed as a synonym without comment (Wozencraft 1993: 331, Berta and Churchill 2012: 219). However, Gray (1849) in fact used this name to describe *Cystophora antillarum*, based on a juvenile male specimen of a Hooded seal, supposedly from Jamaica (Allen 1880, Gill 1866), and the name is not a *nomen nudum*. It is clear that the skin of the Caribbean monk seal that Gray later used to describe *Phoca tropicalis* (Gray 1850), also from Jamaica, was not part of his hypodigm of *antillarum*, to which he explicitly attributed a single *Cystophora* skin and skull (Gray 1849, 1850; Allen 1887:postscript). Thus the name *antillarum* does not correctly belong in the synonymy of *Neomonachus tropicalis* (it is neither a partial synonym nor a *nomen nudum*). It is instead simply a junior synonym for the Hooded seal, *Cystophora cristata* (which occasionally occurs as a Caribbean vagrant: Mignucci-Giannoni and Odell 2001, Ward et al. 2013). The only additional name that correctly belongs in the technical synonymy of *tropicalis* is the junior synonym [*Phoca*] *wilkianus* (Gosse 1851), described from the Pedro Keys, south coast of Jamaica, soon after Gray’s (1850) description of *tropicalis* (see Allen 1880).

##### Etymology.

The new generic name combines the Greek *Neo*- (new), with *Monachus*, the genus name previously used for all monk seals. The name references both the recognition of a new genus within the monk seals and its New World (Western Hemisphere) distribution.

##### Morphological diagnosis.

**Figure 5.**
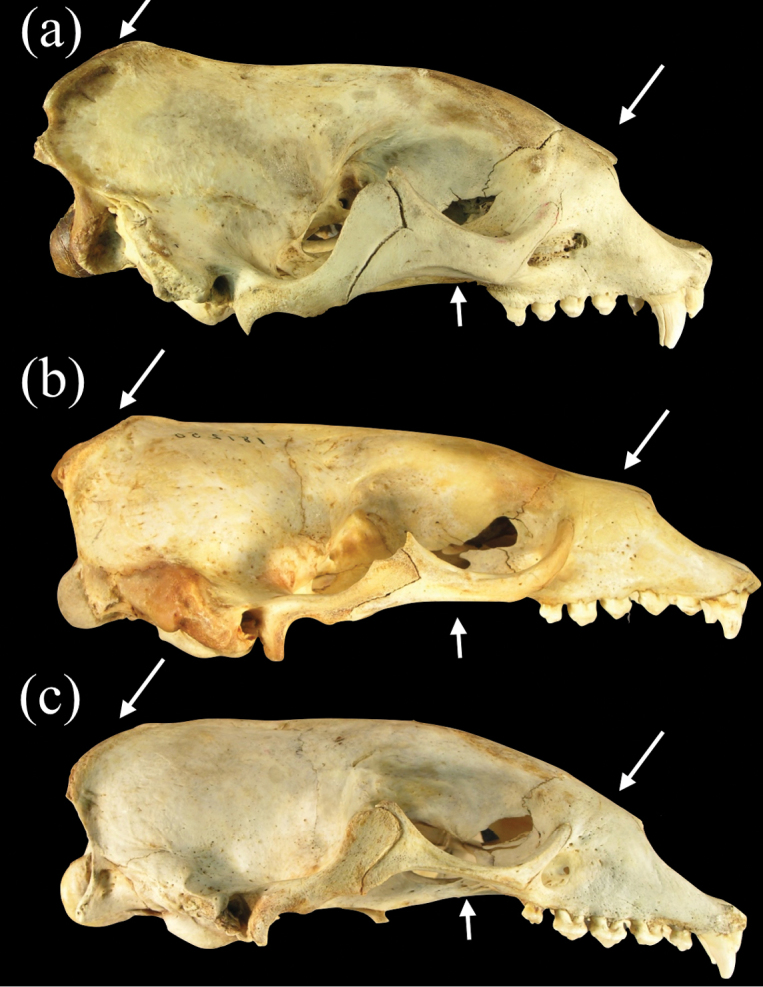
Lateral views of crania of **a** *Monachus monachus* **b** *Neomonachus schauinslandi*, and **c** *Neomonachus tropicalis*. Arrows indicate the more developed occipital crest and zygomatic arches, and deeper snout of *Monachus* compared to *Neomonachus* species.

**Figure 6.**
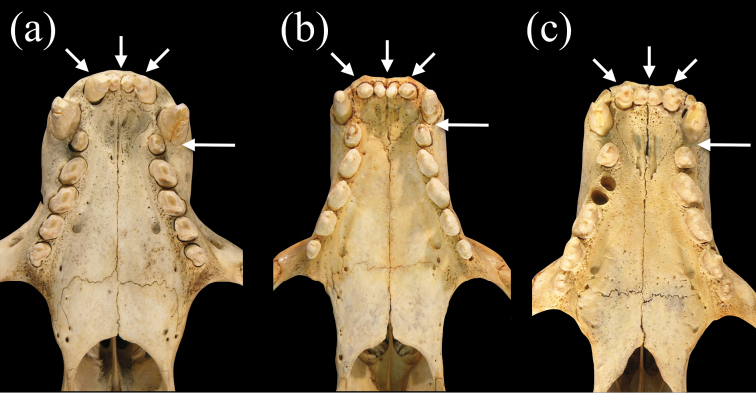
Ventral views of palates of **a** *Monachus monachus* **b** *Neomonachus schauinslandi*, and **c** *Neomonachus tropicalis*. The tooth row of *Monachus* is more crowded, likely as a result of the shorter rostrum, and this results in a more obliquely oriented set of post-canine teeth and the lack of a diastema between the upper canine and the first premolar. In *Neomonachus*, there is a distinct diastema between C1 and P1, and the post-canine teeth are arranged more linearly. The upper incisor arcade of *Monachus* is slightly parabolic due to the posterior placement of the lateral incisors, and the anterior premaxilla appears slightly curved. In *Neomonachus*, the incisor arcade is linear and the anterior premaxilla is straight.

**Figure 7.**
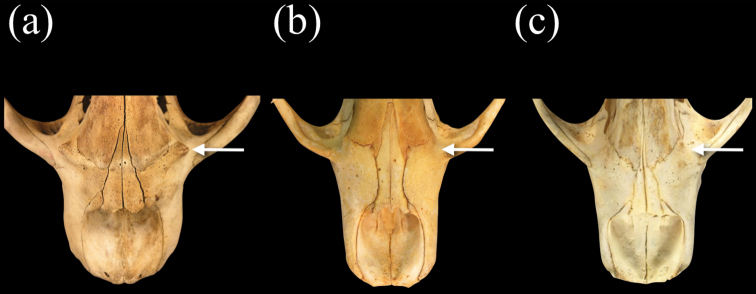
Dorsal view of rostra of **a** *Monachus monachus* **b** *Neomonachus schauinslandi*, and **c** *Neomonachus tropicalis*. *Monachus* exhibits a well-developed antorbital process on the maxilla, immediately inferior to the fronto-maxillary suture. The process is reduced or absent in *Neomonachus*. The nasals of *Monachus* are short and triangular, tapering smoothly posteriorly to produce a point at their union. The nasals of *Neomonachus* are longer and do not taper smoothly.

**Figure 8.**
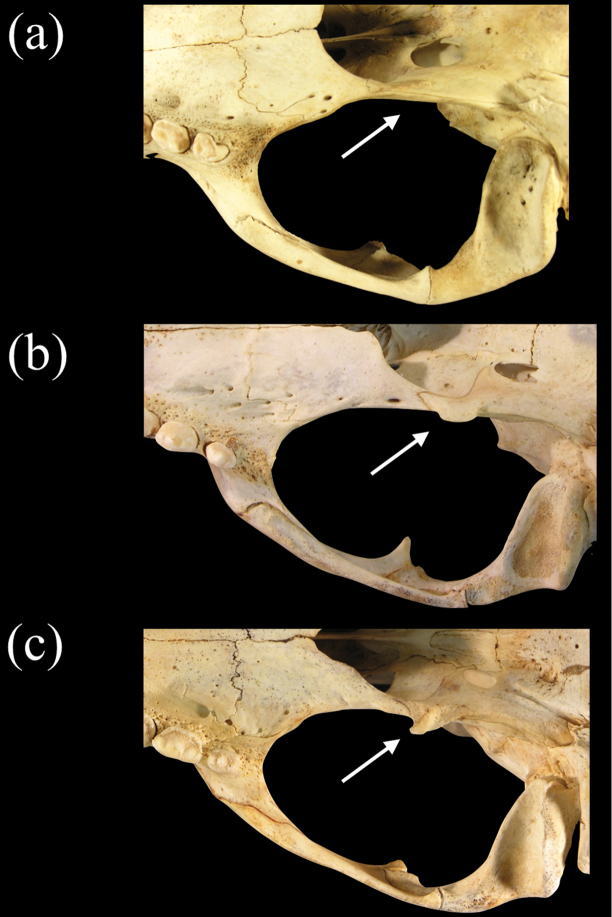
Ventral views of crania of **a** *Monachus monachus* **b** *Neomonachus schauinslandi*, and **c** *Neomonachus tropicalis*, showing the pterygoid region. *Neomonachus* exhibits a well-developed, laterally flared pterygoid hamulus that is visible in dorsal view. The hamulus may be spatulate (*Neomonachus schauinslandi*) or hook-like (*Neomonachus tropicalis*). The hamular process is absent or medially flared in *Monachus*, and is not visible in dorsal view.

Species of *Neomonachus* can be distinguished from *Monachus* in their smaller average body size and in lacking a white ventral patch on the pelage (in both adults and young) (Adam 2004). Species of *Neomonachus* possess a narrower and more gracile skull than *Monachus*, with relatively poorly developed sagittal and occipital crests in even the largest males (Figure 5). The rostrum is low and elongate with a conspicuous diastema between C1 and the first upper premolar (P1). In *Monachus*, the diastema is lacking and the anterior edge of P1 may be positioned medially to the canine (Figures 5, 6). The antorbital process of the maxilla (Figure 7) is present in *Monachus* but is extremely reduced or absent in *Neomonachus* (King 1956). The nasals are relatively narrow and posteriorly extended in *Neomonachus* compared to *Monachus* (Figure 7). The zygomatic arch is dorso-ventrally shallow and the jugal portion lacks a well-developed masseteric margin ventrally or orbital margin superiorly (the zygomatic arch is robust and both margins are well-defined in *Monachus*) (Figure 5). The pterygoid shows a conspicuous, laterally flared hamular process in *Neomonachus* (King 1956) that may be spatulate (*Neomonachus schauinslandi*) or hook-like (*Neomonachus tropicalis*); the process is absent or small and medially inflected in *Monachus* (Figure 8).

**Figure 9.**
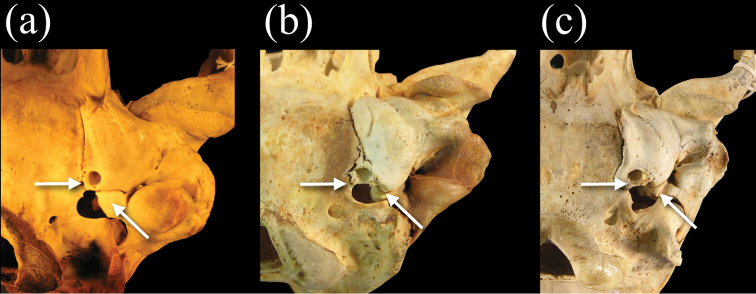
Posteroventral view of the basicranium and left bulla in **a** *Monachus monachus* **b** *Neomonachus schauinslandi*, and **c** *Neomonachus tropicalis*. The bulla of *Monachus* is bordered posteriorly by a ventrally expanded posterior portion of the petro-mastoid complex. The petrosal abuts the bulla’s posterior wall and in ventral view forms the entire lateral and anterolateral border of the posterior lacerate foramen. In *Neomonachus*, the posterior part of the petrosal is visible in the posterior lacerate foramen but remains superior to the bulla. In ventral view, this gives the impression that the anterior border of the posterior lacerate foramen is formed entirely by the bulla. The posterior carotid canal opens posteroventrally in *Monachus*. This apparently results from a relatively complete “ring-like” opening, formed by the bulla. This form of opening is apparent in subadult and juvenile *Monachus*, suggesting that it is not dependent on ontogenetic development or the robusticity of the *Monachus* cranium relative to *Neomonachus*. In contrast, the posterior carotid canal of *Neomonachus* opens directly posteriorly, the opening being an incomplete ring and the dorsal border formed by a flattening of the bulla, perhaps resulting from the bulla’s extension over the petrosal.

In ventral view, the morphology of the petromastoid (petrosal-mastoid) complex in relation to the auditory bulla in *Neomonachus* is diagnostic in comparison to *Monachus*. King (1966) noted that having the petrosal visible within the posterior lacerate foramen was a feature that united *Monachus* and the phocines, although Ray (1976b) pointed out that it is difficult to distinguish the boundaries of the petrosal and mastoid (petromastoid complex) in the posterior lacerate foramen and that this character was far more variable within lobodontines than King had estimated. He argued that a better standard for delineating this character state uniting *Mirounga* and the lobodontines is a bulla in near contact or complete contact with the exoccipital. We agree with Ray’s assessment, but we note that the configuration of the petromastoid complex and bulla with respect to the posterior lacerate foramen is more complex within “*Monachus*” (i.e. *sensu lato*) than has been previously described. In particular, the petromastoid of *Monachus monachus* is clearly ventrally inflated, such that it protrudes below the rim of the posterior lacerate foramen, forming its entire lateral border (Figure 9a). Furthermore, the ventral expansion of the petromastoid almost completely excludes the flat posterior edge of the bulla from the anterior margin of the posterior lacerate foramen. This morphology, which is also present in the fossil taxon *Pliophoca*, has been proposed as synapormorphic for Monachini (Amson and de Muizon 2013). However, in *Neomonachus*, the tapering posterior margin of the bulla lies completely ventral to the petromastoid, the posterior border of which is visible within the neurocranium through the posterior lacerate foramen (Figure 9: b and c). Ray also noted that the posterior carotid foramen opens in full view in *Monachus* (*sensu lato*) but is partially concealed on the medial bulla wall in phocines (Ray 1976b). Our observations show that, for *Neomonachus*, the posterior carotid canal opens directly posteriorly, with a flattened dorsal roof formed by excavation of the caudal entotympanic (Figure 9). In *Monachus*, the canal opens postero-ventrally due to a more complete, ring-like opening. These character-state differences are clearly developed even in juvenile individuals, indicating that they are not the outcome of ontogenetic variation.

**Figure 10.**
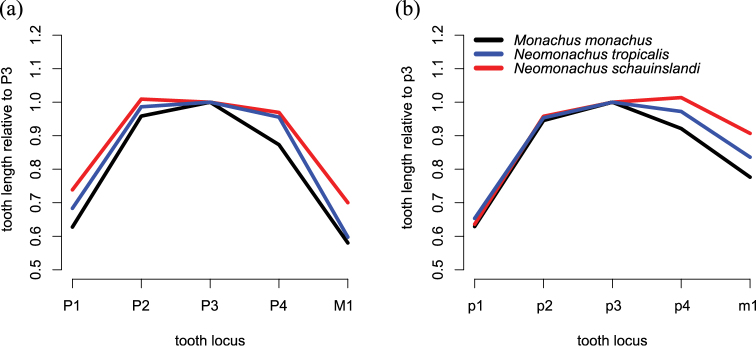
Plots of mean upper (**a**) and lower (**b**) relative post-canine tooth size. Relative tooth size is computed by dividing the mesio-distal length of each tooth by the length of the 3rd premolar (which is typically largest) in the same row.

The upper incisor arcade of *Neomonachus* is sublinear, while that of *Monachus* appears slightly parabolic due to a more posteriorly set I3 (King 1956; Figure 6). The upper post-canine toothrow of *Neomonachus* is arranged more linearly than in *Monachus*, where the teeth are obliquely oriented. In specimens of *Neomonachus* with unworn dentitions, both upper and lower premolars and first molar possess low, blunt central cusps and two posterior accessory cusps, as compared with a high, pointed central cusp and a single posterior accessory cusp for *Monachus*. In both *Neomonachus* and *Monachus*, p3 is the largest of the lower teeth. However, in *Monachus*, lower post-canine tooth size decreases in the order p3, p2, p4, m1, p1, while the lower teeth of *Neomonachus* decrease in the order p3, p4, p2, m1, p1; and p4 may be larger than p3 in some individuals (Figure 10).

**Figure 11.**
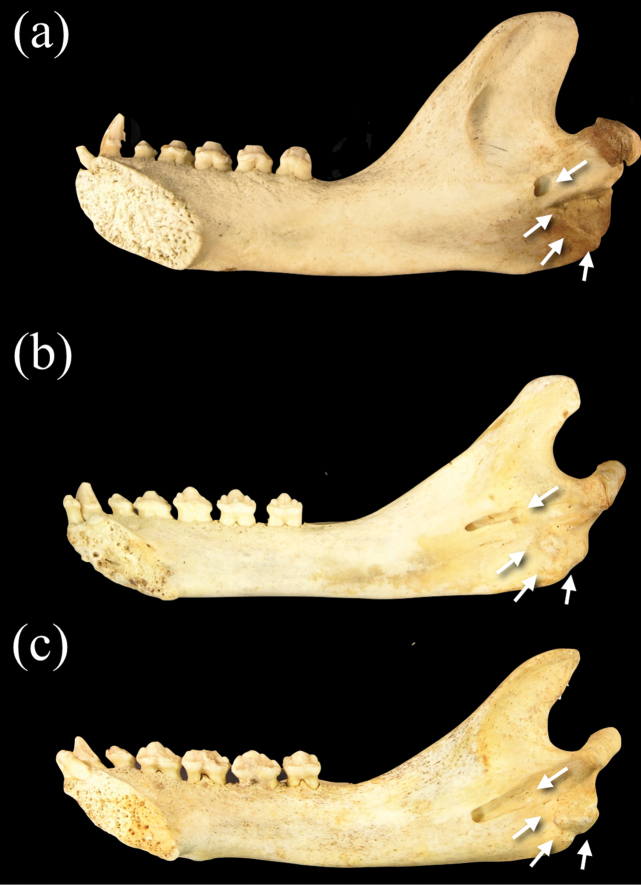
Medial view of right dentaries of **a** *Monachus monachus* **b** *Neomonachus schauinslandi*, and **c** *Neomonachus tropicalis*. The mandibular foramen is situated inferior to the mandibular notch in *Monachus*, and opens immediately to the medial surface of the ramus. In *Neomonachus*, the foramen is anteriorly displaced and is set in a groove or sulcus that extends from inferior to the mandibular notch. Also note the expanded rugose area for insertion of the pterygoid muscles in *Monachus*. This region is poorly developed in *Neomonachus*.

The mandible of *Neomonachus* is long and slender compared with that of *Monachus*, and the coronoid process is lower and less broad antero-posteriorly (King 1956; Figure 11). The mandibular foramen is anteriorly displaced and lies at the termination of a shallow, antero-ventrally oriented sulcus that begins below the level of the mandibular notch. In *Monachus*, the foramen opens directly at this level. The insertion of the pterygoid muscles is relatively undefined in *Neomonachus* as compared to the markedly expanded area evident in *Monachus* (Figure 11).

## Conclusion

We obtained the first DNA sequence data from the recently extinct Caribbean monk seal. Based on phylogenetic analysis and divergence time estimation, we revealed that the Caribbean and Hawaiian monk seals form a well-supported monophyletic New World clade that diverged from the Mediterranean monk seal lineage ~ 6.3 Mya. Our results further implicate the closure of the Central American Seaway in the Late Pliocene as a driver of divergence between the Caribbean and Hawaiian monk seals, supporting a classical hypothesis in pinniped evolutionary biology. In combination, our morphological examinations of museum specimens and our phylogenetic analyses indicate that the substantial evolutionary divergence and trenchant morphological distinctions between the Mediterranean monk seal and the New World monk seals are similar to or greater than levels of molecular and morphological divergence between other sister phocid genera. Because no genus-level name has previously been proposed for the New World monk seals, we name and diagnose a new genus, *Neomonachus*, to accommodate the endangered Hawaiian and extinct Caribbean monk seals, leaving the Mediterranean monk seal as the sole species of *Monachus*.

Our findings and conclusions have broad significance for the two surviving species of monk seal. Because the Caribbean monk seal is already extinct, the elevation of the New World species to a new genus means that both extant monk seals (*Monachus monachus* and *Neomonachus schauinslandi*) are the sole remaining representatives of their respective genera—extremely distinctive seal lineages representing deep, independent evolutionary histories. Both species are critically endangered, with an extant populations of about 1000 individuals for *Neomonachus schauinslandi* and a heavily fragmented and widely distributed population of fewer than 500 individuals for *Monachus monachus*. Formal recognition of two genera for the living monk seals better indicates their true evolutionary, ecomorphological, and taxonomic uniqueness within the context of pinniped evolution, and this taxonomic change grants even greater poignancy to all efforts to conserve these endangered species.

## Supplementary Material

XML Treatment for Neomonachus

## Supplementary material

Supplementary material 1 Amplicons covering *cyt*b in this study. doi: 10.3897/zookeys.409.6244.app1 Data type: Picture Explanation note: Sequence of the *cyt*b gene and the resulting PCR amplicons with length in number of base pairs (bp). Asterisks indicate that the sequence extends over the 3’ or 5’ border of the target sequence. This dataset is made available under the Open Database License (http://opendatacommons.org/licenses/odbl/1.0/). The Open Database License (ODbL) is a license agreement intended to allow users to freely share, modify, and use this Dataset while maintaining this same freedom for others, provided that the original source and author(s) are credited.Dirk-Martin Scheel, Graham J. Slater, Sergios-Orestis Kolokotronis, Charles W. Potter, David S. Rotstein, Kyriakos Tsangaras, Alex D. Greenwood, Kristofer M. Helgen

Supplementary material 2 Alignment of *Neomonachus tropicalis* *cyt*b with extant monk seal *cyt*b sequences. doi: 10.3897/zookeys.409.6244.app2 Data type: Picture Explanation note: The extinct Caribbean monk seal sequence was used as a reference. Dots indicate identity to the reference. Differences are shown as the base change relative to the reference. Numbering starts from the first base of the ATG start codon. This dataset is made available under the Open Database License (http://opendatacommons.org/licenses/odbl/1.0/). The Open Database License (ODbL) is a license agreement intended to allow users to freely share, modify, and use this Dataset while maintaining this same freedom for others, provided that the original source and author(s) are credited.Dirk-Martin Scheel, Graham J. Slater, Sergios-Orestis Kolokotronis, Charles W. Potter, David S. Rotstein, Kyriakos Tsangaras, Alex D. Greenwood, Kristofer M. Helgen

Supplementary material 3 Alignment of three *Neomonachus tropicalis* D-loop hypervariable region sequences (from USNM 100358, 102527, and 102534). doi: 10.3897/zookeys.409.6244.app3 Data type: Picture Explanation note: The *Neomonachus schauinslandi* sequence was used as a reference. Dots indicate identity to the reference. Differences are shown as the base change relative to the reference. Numbering starts at the first base after the primer closest to the 5’ end. The X symbols indicate the break between the two amplicons that are approximately 200 bp apart. Sequences were generated from consensus sequences of 3–5 individual PCR product clones. Several products were cloned and sequenced from more than one amplicon to confirm that the differences observed were not DNA damage related or due to sequencing errors. This dataset is made available under the Open Database License (http://opendatacommons.org/licenses/odbl/1.0/). The Open Database License (ODbL) is a license agreement intended to allow users to freely share, modify, and use this Dataset while maintaining this same freedom for others, provided that the original source and author(s) are credited.Dirk-Martin Scheel, Graham J. Slater, Sergios-Orestis Kolokotronis, Charles W. Potter, David S. Rotstein, Kyriakos Tsangaras, Alex D. Greenwood, Kristofer M. Helgen

Supplementary material 4 Fifty percent majority-rule consensus tree based on 1000 bootstrap pseudoreplicates generated using the maximum parsimony phylogenetic optimality criterion. doi: 10.3897/zookeys.409.6244.app4 Data type: Picture Explanation note: Values at nodes indicate the proportion of bootstrap trees (>50%) for which a particular bipartition was recovered. This dataset is made available under the Open Database License (http://opendatacommons.org/licenses/odbl/1.0/). The Open Database License (ODbL) is a license agreement intended to allow users to freely share, modify, and use this Dataset while maintaining this same freedom for others, provided that the original source and author(s) are credited.Dirk-Martin Scheel, Graham J. Slater, Sergios-Orestis Kolokotronis, Charles W. Potter, David S. Rotstein, Kyriakos Tsangaras, Alex D. Greenwood, Kristofer M. Helgen
