# Supplementary figures and images for: Biogeography and taxonomy of extinct and endangered monk seals illuminated by ancient DNA and skull morphology
Source: Zookeys. 2014 May 14;(409):1–33. doi: 10.3897/zookeys.409.6244 (PMC4042687; doi:10.3897/zookeys.409.6244)

t-RNA-Glu

Cytochrome b

t-RNA-Thr

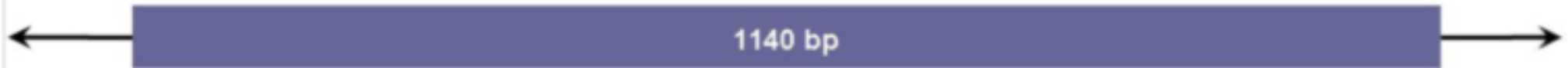

1140 bp

P20  
48 bp\*

P21  
144 bp

P10  
153 bp

P42  
413 bp\*

P7  
149 bp

P22  
135 bp

P23  
77 bp

P32  
131 bp

P16  
549 bp

P40  
229 bp

P24  
109 bp

Supplement: Supplementary material 1 — Amplicons covering cytb in this study. [file zookeys-409-001-s001.pdf]

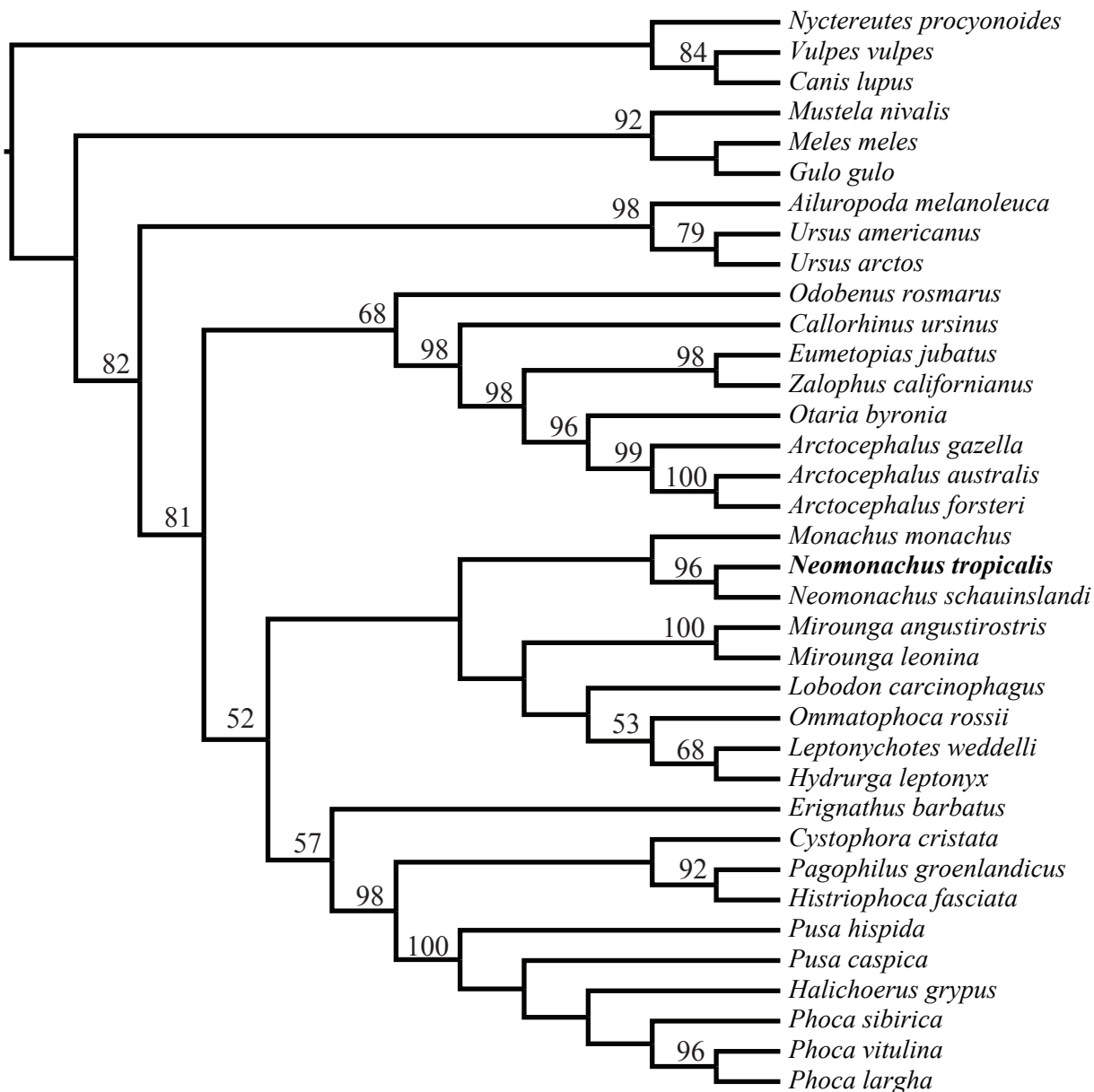

Supplement: Supplementary material 4 — Fifty percent majority-rule consensus tree based on 1000 bootstrap pseudoreplicates generated using the maximum parsimony phylogenetic optimality criterion. [file zookeys-409-001-s004.pdf]
