# Supplementary material for: Biogeography and taxonomy of extinct and endangered monk seals illuminated by ancient DNA and skull morphology
Source: Zookeys. 2014 May 14;(409):1–33. doi: 10.3897/zookeys.409.6244 (PMC4042687; doi:10.3897/zookeys.409.6244)
Supplement: Supplementary material 2 — Alignment of Neomonachus tropicalis cytb with extant monk seal cytb sequences. [file zookeys-409-001-s002.pdf]

```
                    5         15         25         35         45         55         65         75         85         95
N. tropicalis       ATGACCAACA TCCGAAAAAC TCACCCACTA GCCAAAATTA TCAATAACTC ACTCATCGAT CTACCCGCAC CATCAAACAT CTCTGCATGA TGGAATTTTG
M. monachus         .......... .......... .......... ..T.....C. ....C..T.. ......T... .......... .......T.. ...A...... ..A.......
N. schauinslandi    .......... .T........ .......... .....G..C. .......... .........C ........G. .......... ...AAT.... ..A.....C.

                    105        115        125        135        145        155        165        175        185        195
N. tropicalis       GATCCCTCCT CGGAATCTGC CTAATCCTCC AAATTCTAAC AGGCCTATTC CTAGCTATAC ACTACACCTC AGATACAACC ACAGCTTTTT CATCAATCAC
M. monachus         .......... T......... T.......A. .......T.. .......... T......... .T..T..TC. ...C.....T .....C.... .....G..G.
N. schauinslandi    .......T.. A......... .....T..T. ....CT.... .........T .....C.... .......... ...C...... .....C.... ..........

                    205        215        225        235        245        255        265        275        285        295
N. tropicalis       ACACATCTGC CGAGACGTAA ACTATGGCTG AATTATCCGG TACATACACG CTAACGGAGC ATCCATATTC TTTATCTGCT TATATATACA CGTAGGACGA
M. monachus         T........T .......... ....C..... .........A ..T....... .A........ .......... ..C......C ....C..... T..G......
N. schauinslandi    .......... .......... .T..C..... .........A ..T....... .C........ ...T...... ..C....... ....C..... ..........

                    305        315        325        335        345        355        365        375        385        395
N. tropicalis       GGACTATACT ACGGCTCCTA CACATTCACA GAAACATGGA ACATCGGCAT TATCCTCTTA CTTGCCATTA TAGCCACAGC ATTCATAGGC TACGTACTAC
M. monachus         .......... .T........ T......... ........A. ....T..... .......C.G ...A...... .......... .......... ......T...
N. schauinslandi    .......... .T........ T......... ........A. .......... ...T...... ..CA..G.C. .......... .......... ..........

                    405        415        425        435        445        455        465        475        485        495
N. tropicalis       CATGAGGACA AATATCATTC TGAGGAGCAA CCGTCATCAC CAATCTACTA TCAGCAATCC CCTACATTGG AACCGACCTA GTACAATGAA TCTGAGGAGG
M. monachus         .......... ...G...... .......... .T.....T.. ...C...T.. ........T. .......C.. ......T... .......... .T.....T..
N. schauinslandi    .......... ......C... .....G..G. .......... ...C...... .......... .T.....C.. ......T... .......... .......C..

                    505        515        525        535        545        555        565        575        585        595
N. tropicalis       GTTCTCAGTA GACAAAGCAA CCCTGACACG ATTCTTTGCC TTCCACTTCA TTATACCCTT CATAGTAATA GCACTAGCAG CAGTACATCT ATTATTCCTA
M. monachus         A........C .......... ..T.A..... ......C... ........T. .CT....A.. T......TC. .......T.. .......C.. .C........
N. schauinslandi    .......... ..T....... ....A..... ......C..T .....T..T. .......... .......T.. .......... ....C...T. ......T...

                    605        615        625        635        645        655        665        675        685        695
N. tropicalis       CACGAAACAG GATCCAATAA CCCCTCCGGA ATCCCATCCA ACTCAGACAA AATCCCATTT CATCCTTATT ATACAATTAA AGACATTCTA GGAGCTTTAC
M. monachus         .......... ....T..C.. T.....T... ..T....... .......... ...T.....C ..C..A.... .C........ .........G ....CC....
N. schauinslandi    .......... .......C.. T......... ..T....... .......... .........C ..C..A..C. .......... .......... ..........

                    705        715        725        735        745        755        765        775        785        795
N. tropicalis       TCCTTGTTCT AGCCCTAATA CTACTAGTAT TATTCTCACC CGACTTACTA GGAGACCCTG ACAACTATAC CCCCGCCAAT CCCCTAAACA CTCCACCACA
M. monachus         .T..CA.CA. .AT....C.. .......C.C ....T..... .......... ........C. .T.....C.. ...T.....C ..T....G.. .C........
N. schauinslandi    .....A.C.. .ATT...... .........C .......... .......... .......... .......C.T ...T.....C ...T...... ..........

                    805        815        825        835        845        855        865        875        885        895
N. tropicalis       CATCAAACCC GAATGGTACT TCCTATTTGC CTATGCAATC CTACGATCTA TTCCCAACAA ACTAGGAGGA GTCCTAGCCC TAATACTCTC TATCCTAATT
M. monachus         T......... .....A.... .T.....C.. ...C...... ........C. ....T..T.. .......... .......... .......T.. ...T.....C
N. schauinslandi    ...T...... .....A.... .......C.. ...C...... .......... .C.....T.. .......... .......... ..G....T.. C...T.....

                    905        915        925        935        945        955        965        975        985        995
N. tropicalis       CTCGCTATCA TTCCCCTACT CCATACATCA AAACAACGAG GAATAATATT CCGACCTATA AGCCAATGTC TATTCTGACT ATTAGTGGCA GACCTAATTA
M. monachus         .....C.... .......... .......... .......... .......... T.....C..C .......... ........T. .C....A... ......C.C.
N. schauinslandi    .....C..T. .C..T..T.. ...C...... ..G....... ......C... .......... ..T.....C. .......... .....CA... ..........

                    1005       1015       1025       1035       1045       1055       1065       1075       1085       1095
N. tropicalis       CACTAACATG AATCGGAGGA CAACCAGTTG AACATCCCTA CATCACCATT GGCCAACTAG CCTCAATTCT ATATTTCACA ATCCTACTAG TATTAATACC
M. monachus         .......... .......... ........C. ....C..... ...T.TT..C .......... .......C.. ...C.....T .......... ..........
N. schauinslandi    .G........ ...T...... .......... ..T.C..... ..C...T... .......... .......C.. ...C...... ..T.C..... ..........

                    1105       1115       1125       1135
N. tropicalis       CACCATCAGC ATCATCGAAA ACAATATCCT AAAATGAAGA
M. monachus         ..T...T... .......... ...G...... ..........
N. schauinslandi    T.T..C.... ..T....... ....C..T.. ..........
```
