# Supplementary material for: Biogeography and taxonomy of extinct and endangered monk seals illuminated by ancient DNA and skull morphology
Source: Zookeys. 2014 May 14;(409):1–33. doi: 10.3897/zookeys.409.6244 (PMC4042687; doi:10.3897/zookeys.409.6244)
Supplement: Supplementary material 3 — Alignment of three Neomonachus tropicalis D-loop hypervariable region sequences (from USNM 100358, 102527, and 102534). [file zookeys-409-001-s003.pdf]

```
                        |    |     |    |     |    |     |    |     |    |     |    |     |    |     |    |     |    |     |    |
                        5         15         25         35         45         55         65         75         85         95
N. schauinslandi   TTTGGTACTT TTTTAATTTT TAGGGGGGA- AAGCGGTATC ACTCAGCTAT GGCCGTAAAG G-CTCGACGX XXXXXCCCCA TGCATATAAG CATGTACATG
100358             .......... .......... ........GA G.A....... .......... .......... .C.......X XXXXX..... .......... ..........
102527             .......... .......... ........GA G.A....... .......... .......... .C.......X XXXXX..... .......... ..........
102534             .......... .......... ........GA G.A....... .......... .......... .C.......X XXXXX..... .......... ..........
                        |    |     |    |     |    |     |    |     |    |     |    |     |    |     |    |     |    |     |    |
                       105        115        125        135        145        155        165        175        185        195
N. schauinslandi   AACTGGTTGA TTTTACATAA -TGACATATG ATTATGAAAT TGACTTTCAA GGTATAAAAC ACCTATA-GT GGATGCATTT -CACTTAGTC CA-TGAGCCT
100358             .......... .........C ~.......C. .......... .......T.G ......T~.. .A..G..~A. .....T.... ~......... ..A~......
102527             .......... .........C ~.......C. .......... .......T.G ......T~.. .A..G..~A. .....T.... ~......... ..A~......
102534             .......... .........C ~.......C. .......... .......T.G ......T~.. .A..G..~A. .....T.... ~......... ..A~......
                        |    |     |    |     |    |     |
                       205        215        225        235
N. schauinslandi   TGATCACCAG GCCTCGGGAA ATCAGCAACC CTTGT
100358             .........A .......... .......... .....
102527             .........A .......... .......... .....
102534             .........A .......... .......... .....
```
